# Examining the Effects of Sleep Deprivation on Decision-Making: A Scoping Review

**DOI:** 10.3390/bs15060823

**Published:** 2025-06-16

**Authors:** Felix Agyapong-Opoku, Nadine Agyapong-Opoku, Belinda Agyapong

**Affiliations:** 1 School of Medicine, University of Galway, H91 TK33 Galway, Ireland; 2 Department of Psychiatry, University of Alberta, Edmonton, AB T6G 2H5, Canada; nagyapon@ualberta.ca (N.A.-O.); bagyapon@ualberta.ca (B.A.); 3 College of Health Sciences, University of Ghana Medical School, Accra P.O. Box GP 4236, Ghana

**Keywords:** sleep deprivation, decision making, cognitive function, cognitive, impairment, sleep, cognition

## Abstract

Sleep deprivation (SD) is known to impair cognitive functions, and its effect on vigilance and concentration has been explored extensively. However, its effect on the decision-making ability has been researched to a lesser extent. With varying methodologies and conflicting findings in the literature, the effect of SD on decision-making remains complex and inconsistent. Given the critical implications for fields where decision-making is essential, such as medicine, understanding the impact of SD on this cognitive process is crucial. This scoping review aimed to map the existing literature on the effects of SD on decision-making, identify research trends, and highlight inconsistencies to provide implications for practice and research. The review was conducted following PRISMA-ScR guidelines. Databases searched include APA Psych, Web of Science, Scopus, Academic Search Complete, and PubMed. Inclusion criteria focused on peer-reviewed studies from 2014 onward, exploring the impact of SD on decision-making across various tasks and designs. The final selection included 25 articles, representing 2276 participants. The review may suggest that SD, whether partial or total, impairs decision-making ability, with many studies reporting increased risky decisions. The severity of impairment varied based on the type of decision-making task and the duration of SD. However, a few studies reported insignificant effects, particularly in economic decision-making tasks. Moderating factors, such as gender and the origin of sleep loss (voluntary vs. involuntary), were also identified as influential. Sleep deprivation commonly impairs the decision-making ability, with significant implications for high-stakes professions. However, the variability in findings suggests a need for further research into the moderating factors. The review underscores the importance of adequate sleep for cognitive function and the need for policies that mitigate the risks of SD in critical decision-making environments.

## 1. Introduction

Restful sleep, ranging from 7–9 h for adults, is necessary for almost all animals on Earth. It promotes a healthy lifestyle, lowers stress, and can enhance mood and mental health (Hamilton et al., 2007; Minkel et al., 2012; Palmer & Alfano, 2017). Sleep consists of two key stages: REM and non-REM. During REM sleep, the brain is active, dreams occur, and areas related to learning, memory, and emotional processing are stimulated (Hobson & Pace-Schott, 2002; Maquet, 2001; Walker & Stickgold, 2004; Walker & van der Helm, 2009). Non-REM sleep is crucial for physical repair, tissue growth, and immune system strengthening (Besedovsky et al., 2012; Van Cauter & Plat, 1996). A lack of sleep can have a negative impact on memory, alertness, learning, and executive functions (Chee & Chuah, 2008; Jackson et al., 2013; Killgore, 2010; Krause et al., 2017).

Despite the adverse effects of insufficient sleep, research from many studies has demonstrated that at least one-third of adults globally likely do not sleep the recommended minimum of seven hours a night (Ford et al., 2015; Hafner et al., 2017; Hirshkowitz et al., 2015; Watson et al., 2015). This can be due to sleep disorders, like insomnia or chronic sleep syndrome, as well as sleep deprivation.

Sleep deprivation occurs when an individual gets insufficient sleep, whether voluntarily or due to external demands, such as work. Regardless of the cause, it can be harmful to health, as it may increase one’s risk of conditions, such as coronary heart disease, and has been linked to declines in executive brain function (Killgore, 2010; R. Wei et al., 2022). 

One of the facets of executive brain function that has been frequently researched is decision-making. Decision-making is a higher cognitive function that requires multiple regions of the brain to work in unison (collaboration) to render a complex thought, one that evaluates options and selects the most appropriate course of action (Gold & Shadlen, 2007). The hippocampus and the prefrontal cortex are regions of the brain most implicated in decision-making (Saberi Moghadam et al., 2019).

The effect of sleep deprivation on decision-making ability is not linear but rather complex (Jackson et al., 2013). Task impurity is the idea that different decision-making tasks rely on varying combinations of underlying component cognitive processes, such as inhibition or working memory. If sleep deprivation does not impair all these processes to the same extent, then differences in tasks can contribute to inconsistent findings across articles and make it more difficult to confirm an overall effect on decision-making (Jackson et al., 2013).

In some studies, specifically laboratory studies, which are characterized by their immense levels of control, research suggests that the effects of SD on decision-making are inconsistent and sometimes relatively minimal, necessitating further research on the topic (Killgore, 2010; J. Lim & Dinges, 2010). In contrast, in more ecologically valid naturalistic studies, those conducted in real world settings where participants follow mostly normal routines, there are cases where decreases in decision-making ability have been caused by SD (Rosekind, 2005; Samkoff & Jacques, 1991). This would support the idea that adequate sleep is essential for optimal brain functioning and that sleep deprivation can lead to poorer outcomes in tasks requiring high cognitive function.

Some research has suggested that the type of task and whether it is simple and mundane or more complex, logical, and engaging may be effective in determining the effect that SD will have on decision-making ability (Harrison & Horne, 2000). However, inconsistencies in findings across studies, as well as the emergence of newer evidence, highlight the need for an updated review of the literature. While Harrison and Horne’s review provided early insight into this topic (Harrison & Horne, 2000), the field has since expanded significantly, and a more current review is needed to reflect recent evidence and clarify ongoing discrepancies. 

Research over the past decade has focused on many types of decision-making, such as economic, ethical, and probabilistic decision-making (Dickinson & Masclet, 2023; J. Y. L. Lim et al., 2024; Mullette-Gillman et al., 2015), but only a limited number of reviews have concentrated exclusively on specific subtypes, such as the relationship between SD and risky decision-making (X. Wei et al., 2024). 

The findings of the research on the effect of sleep deprivation on decision-making ability have many practical implications, especially in fields, such as medicine. Some evidence has shown that sleep deprivation is associated with increased risky and erroneous decision-making among residents (Aran et al., 2017; Zhang et al., 2021). In professions where the life of an individual is under the responsibility of another individual and where the individual’s decisions can directly affect the safety of others, it is important to investigate any factors that could impair or diminish their decision-making ability. Some doctors and nurses are tasked with working long, mentally demanding shifts, and so the effect of sleep deprivation and fatigue is particularly relevant. Given the high stakes involving such professions as medicine, understanding the impact of sleep deprivation on decision-making is crucial.

This scoping review will aim to map out the existing literature that assesses SD’s role in decision-making to make sense of the existing literature within the last decade, recognize the general research trends, and highlight any inconsistencies. The review will also draw conclusions and provide implications for practice, research, as well as future directions.

## 2. Methods

### 2.1. Protocol and Registration

This scoping review was formulated and conducted in accordance with the Preferred Reporting Items for Systematic Reviews and Meta-Analyses extension for Scoping Reviews (Tricco et al., 2018). There is no published or registered protocol for this scoping review, as details of the systematic search are included in this manuscript.

### 2.2. Eligibility Criteria

The study selection strategy was derived with reference to specific inclusion criteria. The criteria focused on areas of interest, based on the research topic.

Articles deemed eligible for inclusion in this scoping review needed to reference decision-making and how it is affected by a lack of sleep. Experiments randomized controlled trials (RCTs), and all other research designs were accepted. Reviews, dissertations, theses, and protocols were all excluded. Articles were limited to peer-reviewed, quantitative, or qualitative articles that were drafted in English. The year of publication was restricted to papers published from 2014 onwards to ensure that fairly recent articles, which may be more applicable to current practice and context, were captured. 

### 2.3. Information Sources and Search

The search for applicable studies was performed in the following databases using relevant search terms: APA Psych Articles (American Psychological Association Psychological Articles), Web of Science, Scopus, Academic Search Complete, and PubMed.

The search used relevant terms that related to specific concepts, such as sleep deprivation and decision-making. Among these terms were the following: (“Sleep deprivation or Lack of sleep or Sleep deficiency or Insufficient sleep or Sleep shortage or Minimal sleep or inadequate sleep or Sleep deficit or Sleep scarcity”) AND (“Decision making or Deductive reasoning or Decision analysis or Decision formulation or Decision process or Strategic planning or Problem Solving or deductive reasoning”). The database search was completed on 6 January 2025. A sample search result is available in Appendix A.

### 2.4. Selection of Sources of Evidence

Two researchers independently executed the review of the citations during the title, abstract, and full-text screening stages. All subsequent conflicts were resolved after discussion, and a consensus was reached. Of the 43 articles considered eligible for full-text review, 24 were ultimately excluded.

### 2.5. Data Items and Charting Process

Data were gathered by the researchers from each of the selected articles according to the following domains: author (s) name, year of publication, country of study, study design, methods, sample size (N), mean age of sample, and main findings. Data from each included study were validated by two researchers independently to ensure accuracy.

### 2.6. Summary Measures and Synthesis of Results

No quantitative synthesis or statistical summary measures were used. Results were narratively summarized.

This study presents an overview of existing evidence of the role of sleep deprivation on decision-making from the last decade. The relevant data from each included study, including the characteristics and results, is organized and summarized in Table 1. 

## 3. Results

**Figure 1:**
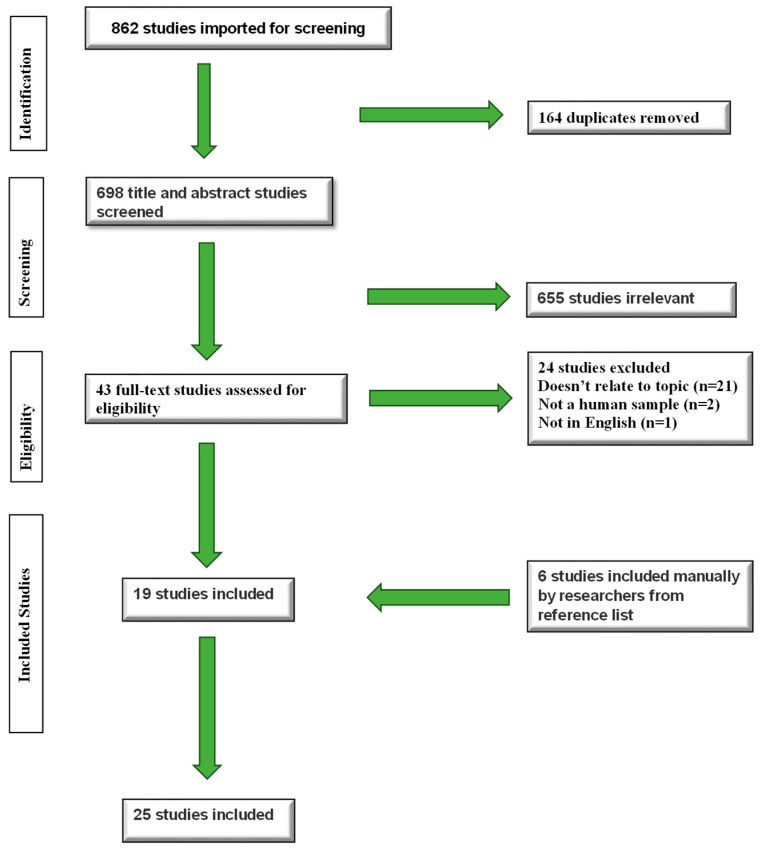
PRISMA flowchart for included and excluded studies.

Of the 862 articles that were imported for screening, 164 of them were identified by the Covidence software as duplicates and automatically removed. Forty-three studies were assessed during full-text screening, which left a total of 19 included articles. Additionally, another 6 articles were found through citation tracking and added manually to the included articles. The PRISMA flowchart in Figure 1 portrays the included and excluded studies.

### 3.1. Summary of Included Studies

Twenty-five articles were included in this review, equating to a total participant count of 2276. The mean ages of the participants ranged from 20.2 ≤ M ≤ 47.6, which makes the population most likely to be representative of an adult population. The study locations were distributed across six continents. Ten of the studies were performed in North America (40%), followed by four in Europe (16%), three in Oceania (12%), six in Asia (24%), one in Africa (4%), and one in South America (4%). 

The studies offered a wide range of designs, broadening the range of data inputs. Many of the studies were of an experimental design (*n* = 19, 76%), one was a case study (4%), one study was a longitudinal design (4%), one was a cross-sectional design (4%), one was a mixed study design (4%), one was a repeated measure observation design (4%), and one was a randomized controlled design (4%). Table 1 gives a summary of the studies included.

### 3.2. Types of Tasks Used to Assess Decision-Making Ability

Decision-making ability is a highly variable aspect to measure, as it may vary depending on the type of task assigned. Different tasks are used to assess different aspects of decision-making. Risk-taking and reward-based decision-making tasks were used in 16 studies. This includes the Iowa gambling task (Brunet et al., 2020; Peng et al., 2022; Schaedler et al., 2018), the Columbia card task (Salfi et al., 2020), the Cambridge gambling task (Santisteban et al., 2019), the balloon analogue risk task (Demos et al., 2016; Lei et al., 2017; Mao et al., 2024; Ruiz-Herrera et al., 2024; Wang et al., 2022), the random lottery pair (Ferrara et al., 2015), the risky-gains task (Lau et al., 2019), lottery choice task (J. Y. L. Lim et al., 2022), Bayes decisions task (J. Y. L. Lim et al., 2024), game of dice task (Xu et al., 2024), and the reversal learning decision task (Whitney et al., 2015). Two articles used complex and realistic decision tasks that provided evidence that was more ecologically valid. These were the Vienna risk-taking test–traffic (Rusnac et al., 2019) and video-based decision-making task (Saddoud et al., 2023). Three studies relied on self-assessment ratings and scales to assess the decision-making ability (Peng et al., 2022; Quan et al., 2023; Vincent et al., 2021). Two studies used delay and temporal discount tasks to assess economic decision-making (Demos et al., 2016; Massar & Chee, 2015). Social decision-making and fairness tasks were used in two studies (Ferrara et al., 2015; Salfi et al., 2020). Gain and loss framing tasks were used in one study (Mullette-Gillman et al., 2015). Six studies also used other decision-making assessment techniques (Bendrick et al., 2016; Demos et al., 2016; Dickinson & Masclet, 2023; Ferrara et al., 2015; Quan et al., 2023; Schaedler et al., 2018). Some of the articles used multiple tasks.

### 3.3. The Empirical Quantification of Effective Fatigue Induction via Sleep Deprivation

Participants may all be subjected to the same period of sleep deprivation; however, the effects of the period on their subjective feeling of sleepiness may differ. A lack of a method to empirically quantify the participants’ level of fatigue could introduce a confounding variable, as well as results that are heavily varied due to participant variability. Different scales and methods are used in these articles to assess the participants’ level of “sleepiness.” Sixteen of the articles used subjective sleepiness scales to standardize the level of fatigue felt by the participants. This includes the Karolinska Sleepiness Scale (Dickinson & Masclet, 2023; Ferrara et al., 2015; J. Y. L. Lim et al., 2022; J. Y. L. Lim et al., 2024; Salfi et al., 2020; Schaedler et al., 2018), the Epworth Sleepiness Scale (Lau et al., 2019; Schaedler et al., 2018), the Pittsburgh Sleep Quality Index (Vincent et al., 2021), the Stanford Sleepiness Scale (Peng et al., 2022; Xu et al., 2024), the Insomnia Severity index (Rusnac et al., 2019), a Visual Analogue Scale-Sleepiness (Santisteban et al., 2019), a fatigue questionnaire (Xu et al., 2024), a subjective sleepiness assessment (Mullette-Gillman et al., 2015), and Likert-type scales (Lei et al., 2017). Eight studies utilized more objective sleepiness and vigilance tests to measure levels of fatigue both directly and indirectly. These are the Psychomotor Vigilance Test (Lei et al., 2017; Massar & Chee, 2015; Mullette-Gillman et al., 2015; Ruiz-Herrera et al., 2024; Wang et al., 2022; Whitney et al., 2015; Xu et al., 2024) and polysomnography (Brunet et al., 2020). Seven articles used other methods or had no measure of fatigue (Bendrick et al., 2016; Demos et al., 2016; Mao et al., 2024; McElroy & Dickinson, 2019; Quan et al., 2023; Saddoud et al., 2023; Xu et al., 2024). Some articles used more than one scale. 

### 3.4. The Effect of the Duration of Sleep Deprivation

The exact number of hours of sleep deprivation, as well as the number of consecutive days that sleep deprivation was administered, varied in some studies. By assessing the specific number of hours and comparing it to the observed effect on decision-making, a correlation may be made. However, due to the difference in decision-making tasks used throughout the articles, it is impossible to create a strict correlation that would describe the exact effect that specific amounts of hours would have on decision-making. Despite this, a general correlation and conclusion can be made in terms of the difference in effect between partial and total sleep deprivation.

### 3.5. Types of Decision-Making Explored

Four of the studies explored more general decision-making ability among the participants (McElroy & Dickinson, 2019; Saddoud et al., 2023; Schaedler et al., 2018; Whitney et al., 2015). Thirteen studies focused explicitly on decision-making under conditions of uncertainty (Brunet et al., 2020; Ferrara et al., 2015; Lau et al., 2019; Lei et al., 2017; J. Y. L. Lim et al., 2022; Mao et al., 2024; Peng et al., 2022; Ruiz-Herrera et al., 2024; Rusnac et al., 2019; Salfi et al., 2020; Santisteban et al., 2019; Wang et al., 2022; Xu et al., 2024). This includes risky decision-making. One study focused on probabilistic decision-making (J. Y. L. Lim et al., 2024). Three studies primarily focused on economic decision-making (Demos et al., 2016; Massar & Chee, 2015; Mullette-Gillman et al., 2015). Three explored occupational decision-making in real-life conditions (Bendrick et al., 2016; Quan et al., 2023; Vincent et al., 2021). One study focused on ethical decision-making (Dickinson & Masclet, 2023).

An increased frequency of risky decisions following sleep deprivation, often demonstrated through tasks, like the balloon analogue risk task or gambling paradigms, was observed in nine of the articles (Brunet et al., 2020; Ferrara et al., 2015; Lau et al., 2019; Lei et al., 2017; J. Y. L. Lim et al., 2022; Rusnac et al., 2019; Salfi et al., 2020; Wang et al., 2022; Xu et al., 2024). Seven studies found impairments in decision-making under conditions of uncertainty (Brunet et al., 2020; Lau et al., 2019; Lei et al., 2017; Peng et al., 2022; Salfi et al., 2020; Wang et al., 2022; Xu et al., 2024), suggesting difficulty in evaluating ambiguous or incomplete information. One study reported deficits in probability-based decision-making (J. Y. L. Lim et al., 2024), reflecting reduced performance on tasks requiring evaluation of known odds. Impairments were also observed in more ecologically valid tasks of real-world decision-making, such as simulated driving and occupational decision scenarios (Bendrick et al., 2016; Rusnac et al., 2019). Additionally, five articles reported declines in general decision-making performance across various domains (McElroy & Dickinson, 2019; Quan et al., 2023; Saddoud et al., 2023; Vincent et al., 2021; Whitney et al., 2015), while one study found sleep deprivation led to more unethical decisions, evidenced by increased cheating behaviors (Dickinson & Masclet, 2023).

## 4. Discussion

Different facets of decision-making have been explored in this review. Sixteen of the twenty-five articles found that sleep deprivation of some capacity, whether partial or total, is associated with reduced decision-making ability (Bendrick et al., 2016; Brunet et al., 2020; Dickinson & Masclet, 2023; Lau et al., 2019; Lei et al., 2017; J. Y. L. Lim et al., 2024; McElroy & Dickinson, 2019; Peng et al., 2022; Quan et al., 2023; Rusnac et al., 2019; Saddoud et al., 2023; Salfi et al., 2020; Vincent et al., 2021; Wang et al., 2022; Whitney et al., 2015; Xu et al., 2024). Across studies, several specific domains of decision-making were affected. These articles provide research that highlights both causal relationships and more real-world examples, providing holistic evidence to support that sleep deprivation deteriorates decision-making ability. 

Three of the studies compared the potentially varying effects of multiple nights of partial sleep deprivation and one night of total sleep deprivation on decision-making (J. Y. L. Lim et al., 2022; J. Y. L. Lim et al., 2024; Salfi et al., 2020). One of the studies found that multiple nights of partial sleep deprivation led to more risky decision-making observed on the Columbia card task and mosaic task, while no significant difference in risk propensity was observed after one night of complete sleep deprivation (Salfi et al., 2020). Another study similarly found that multiple nights of partial sleep deprivation had a more pronounced effect on participants’ decision-making than total sleep deprivation, specifically reducing their ability to integrate multiple sources of information during probabilistic decision-making on Bayes decisions task (J. Y. L. Lim et al., 2024). These findings suggest that the cumulative impact of partial sleep deprivation might be underestimated in real-world scenarios and can be even greater than a single night of total sleep deprivation.

Six of the studies reported an insignificant effect of sleep deprivation on one’s decision-making ability (Demos et al., 2016; Mao et al., 2024; Massar & Chee, 2015; Mullette-Gillman et al., 2015; Santisteban et al., 2019; Schaedler et al., 2018). While three of these articles examined economic decision-making using delay and temporal discounting tasks as well as the gains–losses task (Demos et al., 2016; Massar & Chee, 2015; Mullette-Gillman et al., 2015). These are the “willingness-to-wait” tasks, the “delayed discounting” task, and “the gains choice task and losses choice” task. The evidence presented suggests that sleep deprivation does not have a significant effect on economic decision-making ability as it does for other aspects, such as probabilistic decision-making. For example, Mullette-Gillman et al. (2015) showed that one night of total sleep deprivation only altered the decision-making strategy rather than changing the underlying preferences, such as risk preference on the gains and losses choice task (Mullette-Gillman et al., 2015). Additionally, it did not affect the total information used during decision making. Schaedler et al. (2018) found that individuals with morning or evening sleep restriction of 3 h did not display impairments in decision-making on the Iowa gambling task compared to well-rested participants, suggesting a retained decision-making capacity under condition of uncertainty (Schaedler et al., 2018). Only one other study examined the effect of one night of partial sleep deprivation (sleep reduced by 50%) on decision-making, concluding that it decreased the decision-making ability and led to riskier decisions (Brunet et al., 2020). Notably, both articles used the Iowa gambling task, suggesting that differences in results may be due to methodological factors, such as the length of sleep deprivation and the timing of task administration, or to participant-related factors, such as baseline characteristics. The use of more than one task for each study could allow for triangulation of the results and ensure that the data collected is not task specific. This could enhance the validity of future studies by providing more comprehensive insights. For instance, including an additional task, such as the balloon analogue risk task could help demonstrate the consistency of results across tasks. Studies on the effect of partial sleep deprivation usually focus on the effect of multiple nights rather than a single night (Demos et al., 2016; Dickinson & Masclet, 2023; J. Y. L. Lim et al., 2022; J. Y. L. Lim et al., 2024; McElroy & Dickinson, 2019; Santisteban et al., 2019), leaving a gap in the current literature. More research should be conducted on the effect of a single night of inadequate sleep before informed conclusions can be drawn based on recent evidence. 

Santisteban et al. (2019) found that participants that had multiple nights of 1-h partial sleep deprivation did just as well on the Cambridge gambling task as participants who were well-rested. The other articles which investigated the multiple nights of partial sleep deprivation focused on the effect of 2 h or more, with some reporting a negative influence (Dickinson & Masclet, 2023; J. Y. L. Lim et al., 2024; McElroy & Dickinson, 2019). The findings suggest that multiple nights of 1 h of sleep deprivation may not be sufficient to yield negative effects, although more research into this specific sleep deficit is necessary to support this conclusion.

Other research also supported the notion that sleep deprivation reduced decision-making ability and increased the propensity for risky decisions; however, they highlighted the moderating effect of other factors, such as gender (Ferrara et al., 2015; J. Y. L. Lim et al., 2022). This research also focused specifically on risk in lottery tasks. For instance, Ferrara and colleagues found that following sleep deprivation, males tend to make more risky decisions compared to when they were well-rested, whereas females made fewer risky decisions (Ferrara et al., 2015). Hence, to properly comprehend the relationship between sleep loss and risk propensity, it is essential to consider the influence of demographic and individual differences, such as gender. Males and females might respond differently to sleep loss in risk-related tasks, potentially due to biological or psychological differences. 

One study compared the decision-making ability of individuals in the voluntary sleep deprivation condition to participants in the involuntary sleep loss insomnia condition (Rusnac et al., 2019). The researchers found that the voluntary sleep deprivation condition was associated with increased risky decision-making compared to normal sleepers and people with insomnia. It is important to note that the participants in the study had their long-term sleep times assessed. For instance, participants in voluntary sleep loss conditions had chronic sleep loss. The research demonstrates that the origin of sleep loss does indeed matter, as voluntary sleep loss in the form of sleep deprivation may be more detrimental than involuntary sleep loss.

With regards to ethical decision-making, Dickinson and Masclet (2023) found that after a week of partial sleep deprivation (5–6 h a night) participants made more unethical decisions, reflected by an increase in cheating behaviors (Dickinson & Masclet, 2023). The results of this study are in line with a review on the topic which suggests that sleep deprivation impairs self-regulation and moral awareness, increasing the likelihood of unethical behaviors in the workplace (Barnes & Watson, 2019). Research indicates that sleep deprivation leads to increased impulsivity and a failure to inhibit responses to negative stimuli (Anderson & Platten, 2011), functions which may be necessary for ethical decision-making. Nonetheless, given the limited number of included studies specifically examining ethical decision-making under sleep deprivation, more focused research on this topic is needed to establish more robust and generalizable conclusions.

A few of the articles notably examine the real-world examples of the effects of sleep deprivation on decision-making in professional environments. For example, Bendrick and colleagues focused on the crash of Italia and examined the potential influence of the pilot’s fatigue that led him to make erroneous decisions (Bendrick et al., 2016). Compelling evidence is provided, which portrays real-world evidence of the negative influence of sleep deprivation on decision-making in a real-life context. Additionally, research by Quan and colleagues and Vincent and colleagues investigated how sleep loss affects decision-making in surgeons and sports officials, respectively (Quan et al., 2023; Vincent et al., 2021). Both studies found that sleep deprivation was associated with a reduced decision-making ability in the two contexts. These articles highlight the critical need for individuals, especially those in high-stakes environments, to prioritize sleep as a fundamental component of cognitive health and decision-making competence.

Only one of the articles found that sleep loss was associated with an increased ability to make optimal decisions. Ruiz-Herrera et al. (2024) found that participants portrayed a lower risk propensity after 29.5 h of prolonged wakefulness than during midday before sleep deprivation (Ruiz-Herrera et al., 2024). The strict routine that the participants adhered to, as well as the highly controlled laboratory environment that the participants were in, may have played a role in the results. Although this is a less common finding, it highlights the fact that sleep deprivation does not affect decision-making uniformly and that its effect is not always negative. Nonetheless, almost all the findings report either an increase in risky decision making or no significant effect. The rarity of positive outcomes reinforces the importance of sufficient sleep for preserving cognitive function and supporting rational decision-making.

Since inadequate sleep affects the brain’s ability to function at its highest capacity, the notion that sleep deprivation has detrimental effects on decision-making ability seems to have an understandable biological basis. The prefrontal cortex, a region of the brain involved in decision-making, is particularly sensitive to sleep loss (Krause et al., 2017). When deprived of sleep, this region exhibits reduced activity, leading to impaired judgment and an increased likelihood of making poor decisions. Additionally, a recent study found that changes in the connectivity of the ventromedial prefrontal cortex after sleep loss were associated with increased risky decision making (Wang et al., 2022). Thus, with both biological support, experimental evidence, and real-world examples, it seems appropriate to conclude that sleep deprivation can reliably be associated with decreased decision-making ability. However, the type of decision-making, moderating factors, such as gender and the length of sleep deprivation, are essential when examining possible effects. 

### 4.1. Implications for Policy, Planning and Future Research Directions

The findings from this scoping review underscore the need for policy interventions in sectors where optimal decision-making is critical, particularly in high-stakes environments, such as healthcare, aviation, and transportation. Raising awareness among professionals about the direct impact of sleep on decision-making could lead to more proactive efforts to maintain adequate sleep habits. Research into healthy methods to possibly counter the harmful effects of multiple nights of partial sleep deprivation may be warranted, as it is not always feasible for people to get seven hours of sleep for various reasons.

The review reveals some gaps in current literature, pointing to the need for future research to better understand and mitigate the effects of sleep deprivation on decision-making: First, only a few studies directly compared the effects of different types and durations of sleep loss (e.g., multiple nights of partial sleep deprivation vs. a single night of total sleep deprivation). Future research should address this by systematically varying the duration and intensity of sleep deprivation to determine their specific effects on decision-making. Second, future research on the topic should use more than one decision-making task as this would strengthen the reliability of the evidence and perhaps limit some aspects of participant variability. Having multiple sources of data would allow for a more comprehensive assessment of decision-making processes under sleep-deprived conditions and help identify whether certain types of tasks are more sensitive to sleep loss than others. Third, future studies should explore how individual differences, such as gender, age, personality traits, and resilience to sleep loss, influence decision-making under conditions of sleep deprivation. This could lead to more tailored interventions or guidelines for different populations. Fourth, while most studies examine the short-term effects of sleep loss, there is a lack of research into the long-term cognitive consequences of chronic, cumulative sleep deprivation, and this may be further explored in the future. Fifth, more studies that examine decision-making in real-world settings, such as healthcare, military operations, or finance, are needed to better understand the practical implications of sleep deprivation outside of laboratory environments. Sixth, research on the effect of a single night of partial sleep deprivation is quite limited. Hence, an increased focus on this area would allow for the exact effects to be identified. Considering the various aforementioned gaps in the literature, future research may also focus on conducting a meta-analysis of the literature.

Finally, given the broader implications of impaired decision-making activities such as driving, financial planning, and parenting, future research should support public health initiatives aimed at improving sleep hygiene in the general population. Campaigns could educate the public on the real cognitive costs of sleep loss and promote behavioral changes at a societal level.

### 4.2. Limitations 

This scoping review had a few limitations. First, only the English databases were searched, and only articles written in English were included. This may exclude pertinent studies that were published in different languages, impacting the interpretability of the findings. Second, the sample sizes for most of the studies included in this review were relatively small, under 75, reducing the generalizability of the findings. Third, the review is limited to articles from 2014 onwards, and this may have led to the exclusion of important articles. Finally, individuals under 18 were not included in any of the articles, limiting the ability to generalize the results to the adolescent and child population.

## 5. Conclusions

The effect of sleep deprivation has been explored in several contexts of decision-making, including economic, probabilistic, ethical, and risky decision-making as well as in real world examples. The detrimental effects of sleep deprivation on decision-making abilities are well-supported by the evidence reviewed, but the effect is not unanimous across all studies. The findings across various studies, ranging from experimental designs to real-world scenarios, emphasize the critical role sleep plays in maintaining cognitive function. The evidence not only highlights the general impairment caused by sleep deprivation but also underscores specific vulnerabilities in decision-making processes, such as an increased propensity for risky behavior and a reduced ability to perform complex tasks that require careful judgment.

Moreover, the review reveals that sleep deprivation does not uniformly affect all individuals or decision-making contexts, such as that of economic decision-making. Factors such as the severity and duration of sleep loss, individual differences, such as gender, and the complexity of the decisions at hand can all contribute to varying degrees of impairment. These nuances may suggest that a one-size-fits-all approach may not be sufficient to address the challenges posed by sleep deprivation.

In conclusion, addressing sleep deprivation is a matter of improving individual well-being and a public health imperative that has consequences for safety, productivity, and overall societal function. As the understanding of the relationship between sleep and decision-making continues to evolve, so should the efforts to mitigate the risks associated with insufficient sleep.

## Figures and Tables

**Table 1:** Data accumulated from each study, namely the author (s) name, year of publication, country of study, study design, methods, sample size, mean age of participants, and relevant findings.

Author/Year	Country	Study Design	Methods	Sample Size	Mean Age of Sample	Impact of Sleep Deprivation on Decision-Making	Decision-Makings Test	Measurement of Sleepiness
(Bendrick et al., 2016)	America	Case study	The conditions surrounding the crash of the airship Italia were investigated with the aim of uncovering the possible fatigue-related causes of the crash.	1	N/A	The three erroneous decisions that the captain made that caused the crash were likely a result of his fatigue caused by sleep deprivation.	Ability to Fly the Plane	Excessive Hours of Wakefulness
(Brunet et al., 2020)	Canada	Within-subjects experimental design	Participants completed both the control, well-rested condition, and the partial sleep deprivation condition (one night of 50% sleep deprivation). After each condition, they completed the IGT.	18	23	The duration of REM sleep and deprivation of REM sleep were linked to making riskier decisions on the IGT. This revealed difficulty in decision-making under conditions of uncertainty.	Iowa Gambling Task	Polysomnography
(Demos et al., 2016)	America	Within-subjects experimental design	Participants had four nights of restful sleep (9 h) and four nights of partial SD (6 h). After each sleep condition, participants completed the delayed discounting task, balloon analog task, and go/no-go task.	34	37.0	Multiple nights of partial sleep deprivation were associated with increased impulsive action but not impulsive decision-making.	Delay Discounting Task Balloon Analogue Risk Task Go/No-Go	None
(Dickinson & Masclet, 2023)	America	Experiment	Participants were assigned to either one week of sleep restriction (5–6 h/night) of well-rested sleep (8–9 h/night in which they slept at home. The coin flip task was administered after the fifth night while the two other tasks were given after the seventh night.	237	Not stated	Participants in the sleep restriction condition cheated significantly more than well rested participants in two honesty tasks (coin flip task and matrix task), indicating a decrease in ethical decision-making	Money Burning Task Coin Flip Task Matrix Task	Karolinska Sleepiness Scale
(Ferrara et al., 2015)	Italy	Experiment	Participants performed two sessions. In one session, they had a restful night of sleep before performing a decision-making task. In the second session, participants underwent a night of total sleep deprivation before doing the task.	32	24	Following sleep deprivation, males tended to make riskier decisions than when they were well-rested, whereas females became more cautious.	Random Lottery Pair Linear Dictator Game	Karolinska Sleepiness Scale
(Lau et al., 2019)	China	Longitudinal design	Participants underwent a 7-day protocol with assessments of sleep and risk-related decision-making at baseline (T1) and again 12 months later (T2). Participants completed a Consensus Diary to measure sleep debt, sleep time, sleep need, and other sleep-related details.	166	Not stated	Individuals with high sleep needs but short sleep duration had an increased propensity for riskier decisions.	Risky Gains Task	A Chinese version of the Epworth Sleepiness Scale
(Lei et al., 2017)	China	Within-subjects, repeat-measure, experimental design	Participants underwent two counterbalanced experimental sessions, rested wakefulness (normal sleep routine) and total sleep deprivation (36–40 h), separated by at least 2 weeks. Decision tasks were conducted during both sessions, along with regular assessments of sleepiness, mood, and alertness. MRI scans were taken after each condition.	32	23.1	The results showed that sleep deprivation increased activation related to risk modulation in the left inferior frontal gyrus, and this increased activation was positively correlated with higher risk-taking propensity after sleep deprivation. In sleep-deprived participants, activation in the ventral striatum and thalamus increased during cash-out events, while activation in the middle temporal gyrus decreased following explosions (i.e., loss of money), adding evidence for greater risk propensity following SD.	Balloon Analogue Risk Task.	Psychomotor Vigilance Task Likert-type Rating of Sleepiness
(J. Y. L. Lim et al., 2022)	Australia	Experiment	Participants were either assigned to the well-rested (9 h time in bed for 6 nights) and total sleep deprivation (1 night) protocol or the WR and sleep restricted (4 h time in bed for 4 nights) protocol. After a night of WR, SD, or SR, participants completed the lottery choice task. The risk of choices was analyzed afterwards.	47	24.57	In the gains-only model, Males did not change risk preference during either TSD or SR, while females became significantly more risk-averse during both. In the LOSSES only model, female participants did not change their risk preference after TSD, while male participants became significantly more risk-seeking after TSD than WR.	Lottery Choice Task	Karolinska Sleepiness Scale
(J. Y. L. Lim et al., 2024)	Australia	Experiment	Participants all completed a well-rested condition and one of two randomly assigned experimental conditions: SR, which involved four hours’ time in bed for four nights or one night of TSD. After each condition, they completed the Bayes decision task.	46	24.76	Participants in the first experiment demonstrated a decreased reliance on both information sources when making probabilistic decisions following sleep restriction. Sleep restriction reduced the integration of multiple information sources during decision making. No significant change was observed after TSD	Bayes decisions task	Karolinska Sleepiness Scale
(Mao et al., 2024)	America	Within-subjects experimental design	Participants completed an fMRI scan after one night of TSD and another scan after RW (8 h’ time in bed) in counterbalanced order. A decision-making task was performed during each scan.	56	32.59	Sleep deprivation did not alter participants’ risk-taking behavior but significantly reduced brain activity in regions associated with processing wins and losses, including the anterior cingulate cortex, insula, and putamen. Risk induced activation of the insula was negatively correlated with risk propensity during RW but not TSD.	Balloon Analogue Risk Task (modified)	None
(Massar & Chee, 2015)	Singapore	Experiment	Participants completed a willingness-to-wait task either when they were well-rested or following a night of complete sleep deprivation.	29	22.28	Sleep deprivation and decreased vigilance did not affect participants’ ability to make decisions in the adjustment of waiting times.	Willingness-to-Wait Task	Psychomotor Vigilance Test
(McElroy & Dickinson, 2019)	America	Mixed study design	There were three conditions that the participants were assigned to: a control, a 1-week partial sleep deprivation (5–6 h), or a well-rested (9 h) condition. There was also either a circadian match or a mismatch condition. Decision-making tasks were completed afterwards.	140	21.9	Participants in the SD circadian mismatched condition performed more poorly on only one decision-making task compared to the well-rested condition.	Complex Decision Tasks	None
(Mullette-Gillman et al., 2015)	Germany	Experiment	All participants completed both a TSD session and a restful wakefulness session. In each session, all participants completed a gains choice task, a losses choice task, and a loss aversion task. Choice strategy, uncertainty preference, and decision-making ability were evaluated.	29	21.66	One night of SD alters the information the participants rely upon to make their choices. Sleep deprivation alters a person’s decision-making by altering the informational strategies that participants use. After sleep deprivation, participants relied less on complex, maximizing information and more on simpler, satisfying information. Sleep deprivation shifted the decision-making strategy rather than changing the underlying preferences.	Gains Choice Task Losses Choice Task	Psychomotor Vigilance Test and Subjective Sleepiness Assessment
(Peng et al., 2022)	China	Experiment	Participants performed in both daytime and nighttime shifts conditions. In the first condition, the night shift group completed the fatigue assessment test, A-DMC, and IGT. The second condition was that the participants completed these tests after a daytime shift.	107	25.33	The IGT scores and decision-making abilities of nurses significantly deteriorated after working a night shift (mentally fatigued), with a clear connection between the decline in decision-making competence and their performance on the IGT.	Iowa Gambling Task Adult Decision-making Competence Scale	Stanford Sleepiness Scale
(Quan et al., 2023)	America	Repeated measures Observational design	Surgeons were studied prospectively using direct observation and self-reported data during post-call (>2 h of nighttime clinical duties) and non-post-call.	60	47.6	Non-technical skills for surgeons’ ratings demonstrated poorer performance for decision-making while on post-call. Fewer hours of sleep were also related to lower ratings for decision-making.	Performing Surgical Duties Self-reported Decision-making Ability on a 4-point Scale	None
(Ruiz-Herrera et al., 2024)	America	Within-subjects, repeated measures experimental design	Participants were allowed 9 h of sleep for the two nights before undergoing 39 h of total sleep deprivation. Vigilance and decision-making tasks were administered every 2 hours on the second day (baseline) for a total of 7 times, and on the third and fourth days (constant routine) for a total of 19 times.	13	26.46	In the balloon analogue risk task, participants showed a greater risk-taking propensity around midday before sleep deprivation, and a reduced risk-taking tendency after prolonged wakefulness (29.5 h awake).	Balloon Analogue Risk Task	Psychomotor Vigilance Task
(Rusnac et al., 2019)	France	Quasi-experimental design	Participants in the voluntary sleep loss, normal sleep, and insomnia groups were shown videos of dangerous traffic scenarios from a 1st person POV. The participants assess and react while the researchers measure the risk propensity.	536	22.25	Participants with voluntary sleep deprivation (around 2 h) made more risky decisions in virtual driving situations compared to normal sleepers and participants with insomnia.	The Vienna Risk-Taking Test–Traffic	Insomnia Severity Index
(Saddoud et al., 2023)	Tunisia	Experiment	Participants were assigned to either the sleep deprivation condition (One night) or the well-rested condition. They then completed a series of tasks, including a decision-making task.	24	20.2	The total sleep deprivation (TSD) condition led to reduced decision accuracy and increased decision time compared to the normal sleep (NS) condition.	Video-based Decision-making Task	None
(Salfi et al., 2020)	Italy	Experiment	Exp 1: Participants were tested for decision-making ability after one night of regular sleep and one night of complete sleep deprivation. Exp 2: Participants were tested after five nights of restful sleep and five nights of partial sleep deprivation.	32 42	22.13	A single night of complete sleep deprivation did not significantly impact data gathering to make decisions or risky decision making. Multiple nights of partial sleep deprivation increased impulsive and risky decision-making.	Mosaic Task Columbia Card Task	Karolinska Sleepiness Scale
(Santisteban et al., 2019)	Canada	Randomized controlled between-subjects experimental design	A 6-night baseline sleep assessment was completed at home by each participant using actigraphy. Participants were then randomly assigned to either a sleep restriction group (1 less hour of sleep for 6 nights) or a placebo group exposed to non-therapeutic light, while maintaining normal sleep patterns. Cognitive testing and sleepiness questionnaires were completed throughout the week.	93	Not stated	Performance on the decision-making task was not significantly affected by partial sleep deprivation, reflected by similar performances between the two conditions.	Cambridge Gambling Task	Visual Analogue Scale-Sleepiness
(Schaedler et al., 2018)	Brazil	Experiment	Participants were assigned to the normal sleep condition, morning sleep restriction condition (3 h less sleep), or evening sleep restriction condition (3 h less sleep). Afterward, they completed various tasks to assess their cognitive ability.	48	Not stated	Compared to the control group, individuals with either morning or evening sleep restriction did not exhibit any significant impairments in decision-making.	Iowa Gambling Task Go-NoGo Test Stroop Test	Epworth Sleepiness Scale Karolinska Sleepiness Scale
(Vincent et al., 2021)	Australia	Cross-sectional survey design	Sports officials (*n* = 371) completed an online questionnaire assessing self-reported sleep quantity and quality on regular nights, before and after competitions, and their perceived decision-making.	371	37.2	Self-reported reduced sleep had a negative impact on perceived decision-making abilities.	Self-rating on a Likert scale.	Pittsburgh Sleep Quality Index
(Wang et al., 2022)	China	Within-subjects experimental design	Participants completed two fMRI sessions: one following a night of normal sleep and another after 36 h of total sleep deprivation. Sleep and wakefulness were closely monitored. After each scan, participants performed a vigilance test, a risk-taking decision-making task, and an alertness assessment.	21	23.48	A single night of sleep deprivation led to a reduction in functional connectivity between the ventromedial prefrontal cortex (vmPFC) and thalamus bilaterally, alongside an increase in connectivity between the vmPFC, both the dorsolateral prefrontal cortex (dlPFC) and the parietal lobe. Greater risk-taking was linked to stronger vmPFC–dlPFC connectivity and weaker vmPFC–thalamus connectivity. These findings demonstrate that a lack of sleep alters brain connectivity in ways that predict the increased risk-taking behavior found after SD.	Balloon Analogue Risk Task	Psychomotor Vigilance Task
(Whitney et al., 2015)	America	Randomized controlled design	Participants were either assigned to the sleep-deprived condition (62-h sleep deprivation) or the control condition (no SD). The participants completed the decision task, and their abilities were assessed.	26	Not stated	Sleep deprivation decreased the decision-making ability of the participants during the task.	Reversal Learning Decision Task	Psychomotor Vigilance Test
(Xu et al., 2024)	China	Repeated measures experimental design	The study involved healthy male college students in Beijing who underwent 36 h of total sleep deprivation, during which they were repeatedly assessed at seven time points.	37	23.18	Sleep deprivation led to an increased tendency for risky decision-making, with the effects often appearing 15–20 h after SD. Their ability to use negative feedback was impaired after 8 h of SD.	Game of Dice Task	The Stanford Sleepiness Scale Fatigue Questionnaire Psychomotor Vigilance Test

## Data Availability

No new data was generated.

## Appendix A

Scopus

1. (“Sleep deprivation” or “Lack of sleep” or “Sleep deficiency” or “Insufficient sleep” or “Sleep shortage” or “Minimal sleep” or “inadequate sleep” or “Sleep deficit” or “Sleep scarcity”).mp. [mp=title, abstract, heading word, table of contents, key concepts, original title, tests & measures, mesh word]: 14,148

2. (“Decision making” or “Deductive reasoning” or “Decision analysis” or “Decision formulation” or ‘Decision process” or “Strategic planning” or “Problem Solving” or “deductive reasoning”).mp. [mp=title, abstract, heading word, table of contents, key concepts, original title, tests & measures, mesh word]: 807,276

3. 1 and 2: 403
